# Proposal for a Fitness Program in the School Setting during the COVID 19 Pandemic: Effects of an 8-Week CrossFit Program on Psychophysical Well-Being in Healthy Adolescents

**DOI:** 10.3390/ijerph18063141

**Published:** 2021-03-18

**Authors:** Stefania Cataldi, Vincenzo Cristian Francavilla, Valerio Bonavolontà, Ornella De Florio, Roberto Carvutto, Michele De Candia, Francesca Latino, Francesco Fischetti

**Affiliations:** 1 Department of Basic Medical Sciences, Neuroscience and Sense Organs, School of Medicine, University of Bari “Aldo Moro”, 70124 Bari, Italy; stefania.cataldi@uniba.it (S.C.); o.deflorio@studenti.uniba.it (O.D.F.); roberto.carvutto@uniba.it (R.C.); michele.decandia@uniba.it (M.D.C.); francesca.latino@uniba.it (F.L.); francesco.fischetti@uniba.it (F.F.); 2 School of Engineering, Architecture, and Motor Sciences, Kore University of Enna, 94100 Enna, Italy; vincenzo.francavilla@unikore.it

**Keywords:** high-intensity training, self-efficacy, physical fitness, physical exercise, pandemic COVID 19

## Abstract

Background: The Italian government promoted social distancing, in which the suspension of any social event, suspension of all activities practiced in gyms, sports centers and their closure was ordered. The social distancing in the school environment and the use of strategies to limit viral infection are not very compatible with group motor activity and team sports. The aim of this study is to verify the effectiveness of a CrossFit program in order to mitigate the deficits in fitness caused by COVID-19 prevention measures and to evaluate the effects on self-efficacy in a group of young adolescents. Methodsː 30 healthy participants were randomly allocated into an intervention group (IG) that performed the 8 weeks CrossFit training program or control group (CG). Physical fitness tests (i.e., Squat, push-up, lunge, and 20 m run) and psychological measures Regulatory Emotional Self-Efficacy scale (RESE) were performed at baseline and after 8 weeks. Resultsː After 8 weeks, the intervention group showed significant improvements for all fitness tests (*p* < 0.0001). Additionally, higher scores for the RESE negative and positive (*p* < 0.0001) scales were found in the intervention group. No statistical differences were found in the control group except for the push up test. Conclusionsː the 8-week CrossFit intervention program could positively affect the general physical well-being and improve the emotional perceived self-efficacy in healthy adolescents.

## 1. Introduction

The coronavirus disease 2019 (COVID-19) pandemic caused an unprecedented crisis, influencing the lives of millions of people worldwide [1]. The Italian government promoted social distancing, in which the suspension of any social event, suspension of all activities practiced in gyms, sports centers and their closure was ordered [2,3]. In the last year, some studies have shown the effects of the social-isolation period and home confinement. Social isolation creates various effects in different domains: physical activity, weight gain, psychological states, mood disturbances, anxiety, depression and irritability [4,5,6,7,8]. The adverse psychological effects are associated with a reduction in physical activity and increase in incorrect eating habits [9]. In this particular juncture marked by the COVID-19 pandemic, the school continues its work of teaching students but also of promoting health and physical activity. However, social distancing in the school environment and the use of strategies to limit viral infection are not very compatible with group motor activity and team sports. Some fitness programs can be a solution; an exercise program such as CrossFit can respect social distancing in the school gyms [10,11,12]. This exercise program requires minimal equipment and has the potential to align with physical education and sport objectives in secondary schools. It is well known that High-Intensity Interval Training, such as CrossFit, positively influences physical and mental well-being [12,13]. Although regular exercise has been shown to improve well-being and mood state, the relationship between exercise and well-being is complex as it is influenced by intensity and duration of exercise as well as by overtraining and exercise results [14,15]. Emotions and mood states are known to be influenced by the modality and intensity of an exercise session [16]. It is also well known that high-intensity interval training (HIIT) positively influences physiological and psychological well-being [17]. CrossFit is a form of high-intensity interval training based on functional movements that has seen a rapid spreading since its inception in 2000 and it is nowadays recognized as one of the fastest growing modes of HIIT [18,19]. Previous research on CrossFit has focused on aerobic fitness and body composition, on motivational variables, on psychological concomitants, on culture and on the use of music in CrossFit, on using CrossFit as a sport education model for secondary school students, on improving health-related fitness in adolescents and on issues related to injury and safety [20,21,22]. Despite a certain number of studies, scientific data regarding the practice of CrossFit are poor and yet few studies have examined psycho-social factors in relation to adherence [17,23]. The CrossFit Teens™ program was designed specifically for improving fitness and resistance training skill competency in adolescents (ages 12–18 years) and incorporates combinations of nine core strength exercises in a group training setting [10,24]. In 2006, Eather et al. showed a CrossFit program administered to adolescents. This study demonstrated that CrossFit is feasible and efficacious for improving health-related fitness in adolescents [25]. Character strengths are among the most investigated individual characteristics in the field of positive psychology [26]. Therefore, character strengths can be seen as useful individual characteristics in protecting mental health (reducing symptoms of distress and increasing self-efficacy) following a pandemic too. Self-efficacy is able to explain various cognitive and motivational aspects related to learning, including the impact of positive experiences and successes, perseverance in commitment, optimism and the development of interests in specific cultural and professional fields [27]. Self-efficacy refers to the beliefs that an individual is capable to adequately manage situations and to master activities in order to achieve the desired results. These personal beliefs represent an important factor that can influence different areas of experience, including the school context. The aim of this study is to verify the effectiveness of a CrossFit program in order to mitigate the deficits in fitness caused by COVID-19 prevention measures and to evaluate the effects on self-efficacy in a group of young adolescents. The main novelty of this study, compared to the current literature, lies in having demonstrated the effectiveness of cross-fit as an adapted physical activity to the pandemic period. As described, the proposed program can be carried out and taught, by academically qualified personnel, with an individualized method in conditions of social distancing, in complete safety. In addition, the study broadens the observation to the psychological variables, related to the spaced CrossFit training program, which are most affected by the pandemic period (emotional regulation and self-efficacy), demonstrating a clear improvement.

## 2. Materials and Methods

### 2.1. Subjects

Thirty subjects were enrolled for this study (18 males; 12 females; Age: 18.26 ± 0.52 years; Weight: 66.56 ± 10.91 Kg; Height: 171.13 ± 7.09 cm). Data collection started in June 2020 and ended in August 2020 after eight weeks of intervention. 

### 2.2. Experimental Design

**Figure 1:**
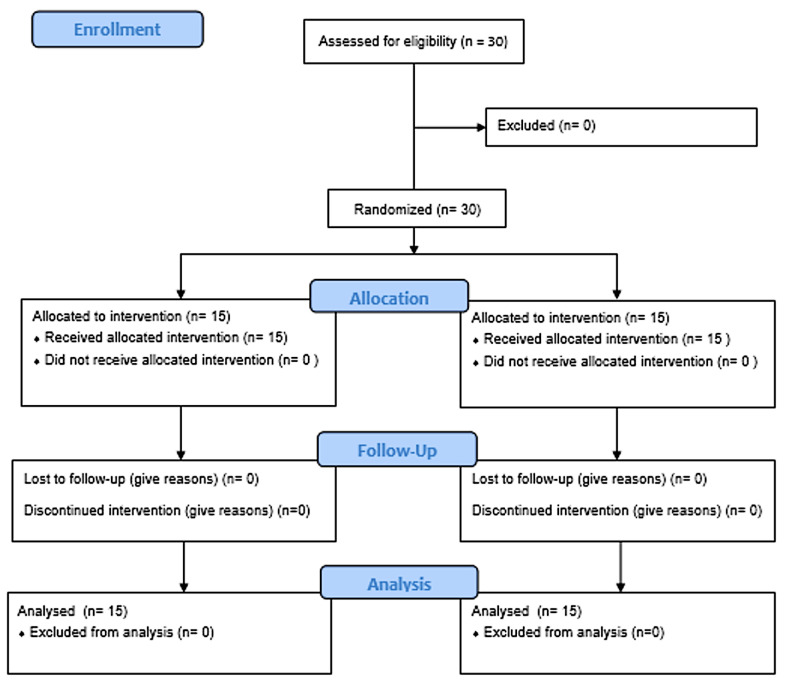
Flowchart of the study.

Our study was carried out in compliance with the principles of the Italian Data Protection Act (196/2003) and the Declaration of Helsinki. Written informed consent was obtained from each subject before the participation. The study was inserted in the Adapted Physical Activity Prevention Program, which had obtained Ethical Approval (assigned number 553/EC). Before study participation, informed consent was provided by each participant. All participants were treated in agreement with the ethical guidelines of the American Psychological Association with respect to consent, confidentiality and anonymity of the answers. All participants were recruited from the University of Bari Sports Lab. The IG and the CG were recruited from subjects practicing regular and traditional physical exercise for at least one year (pre-pandemic period COVID-19). Their pre-pandemic training regime consisted of two weekly sessions with the same volume of load expected for the period t^0^ < t^1^ of the experiment. Respecting the male/female ratio, the subjects were divided using a 1:1 randomization strategy into two groups: the first group was the intervention group (IG; n:15) and the second was the control group (CG; n = 15). The allocation sequence was computer generated, with group allocation directed by a research assistant who did not participate in the study (Figure 1). The Consolidated Standards of Reporting Trials (CONSORT) Statement was set as a standard [27]. The groups were comparable in terms of age. The group characteristics are reported in Table 1. All measurements were performed twice, and the arithmetic mean was recorded for evaluation [28]. IG group performed the CrossFit program, described in detail in the intervention section, while CG group was formed by participants of a general functional training program; the CG carried out a non-standardized and equipment-free exercise program that included a sequence of stretching exercises and aerobic exercise (160 min of moderate-intensity aerobic activity each week). 

#### 2.2.1. Method of Testing

First, anthropometric measurements were collected. Body height (in cm to the nearest 0.1 cm) was measured using a SECA^®^ stadiometer (Hamburg, Germany), and body weight (in kg to the nearest 0.1 kg) was measured using Tanita^®^ digital scalesO (Ascoli Piceno, Italy) [29,30]. Abdominal circumference (in cm to the nearest 0.1 cm) was measured at the level of the greatest anterior extension of the abdomen in a horizontal plane, when the subject stood [30].

#### 2.2.2. Fitness Battery Test 

The physical fitness was assessed through squat test, lunge test, push-up test, lunge test, 20 m shuttle-run test, all performed to exhaustion. The squat test (ST), lunge test (LT) and the push-up test (PUT) were used to measure lower (ST, LT) and upper-body (PUT) muscular strength and endurance. For the ST, LT and PUT, participants were instructed to perform consecutively as many squats, lunges and push-ups as possible [31,32]. The score was the number of successfully completed push-ups [33]. The 20 m shuttle run test (SRT) was used to assess aerobic fitness [34]. Participants were instructed to cover a set distance of 20 m as many times as possible. The score was the number of successfully laps run.

#### 2.2.3. Regulatory Emotional Self-Efficacy Scale (RESE)

RESE was developed to assess self-efficacy in regard to emotional regulation and, in particular, perceived self-efficacy in managing a negative response to adversities or frustrating events and in expressing or managing positive emotions such as joy, enthusiasm, and pride [27]. The scale of perceived self-efficacy in the management of negative emotions is made up of 8 items, while the scale of self-efficacy perceived in the expression of positive emotions is made up of 7 items. For each item, subjects evaluate the degree to which they believe they are able to regulate negative emotions and the degree to which they believe they are able to express their positive emotions, using a 5-point scale. 

#### 2.2.4. Protocol CrossFit Program 

The CrossFit fitness program was designed specifically for adolescents. In 2016, Eather et al. used this program in an Australian secondary school [25]. The results of the study showed that the program was successful in significantly improving body composition and levels of fitness. The authors did not aim to evaluate psychological and self-efficacy implications, but it was found that students were highly satisfied with the program. In addition, recruitment and retention levels were high, and adherence to the program was excellent [25]. Evaluating all this, we decided to use the same CrossFit program with some changes on the use of equipment that do not comply with the anti-COVID-19 prevention measures. In addition, the fitness program was administered in the summer of 2020, in an outdoor location and respecting the distance of two meters between the subjects, and regular and careful surface sanitation of everyday objects and gym equipment was performed [35]. IG participants carried out the CrossFit program twice a week for 8 weeks, and each training session lasted 60 min. The program was led by a Bachelor in Motor Sciences with a technical qualification issued by one of the Italian Associations for the teaching of CrossFit. According to CrossFit methodology, males and females underwent the same protocol of exercises and each exercise was not performed for longer than 30 s in order to minimize any risk of exhaustion and fatigue. For each of the sessions, participants train with same-level peers with personal intensity according to their levels of fitness and of physical abilities, as reported by previous research [36]. Each session was composed by a 10-min dynamic warm up, followed by 10 to 20 min of technical drills, by a workout of the day (WOD = 10 to 20 min) and, finally, by a cool down with stretching exercises (5 to 10 min) plus 10 min at the beginning to set up the session. Table 2 shows the specific content of each training session in detail.

#### 2.2.5. Statistical Analysis

All numerical data were entered on an Excel sheet before being analysed. Means scores and standard deviations (*s*) for pre-test (T_0_) vs. post-test (T_1_) intervention evaluations were calculated separately for the IG and CG group. The paired *t*-test (*p* < 0.05) for independent variables was used to detect significant differences in the groups’ performance (IG vs. CG). Descriptive statistical analysis was performed using the mean ± SD and a 95% confidence interval. To estimate the effect, Cohen’s d was calculated. Cohen’s guidelines, whereby a value of 0.2 denotes a small, 0.5 a medium, and 0.8 a large effect size [36,38]. Statistical analysis was performed using StatSoft’s STATISTICA software (Windows, version 8.0; Tulsa, OK, USA) and GraphPad Prism software (Windows, version 5.0; La Jolla, CA, USA).

## 3. Results

Thirty subjects were analyzed in this study. The flow of participants through the study process is displayed in Diagram 1. The anthropometric data of the two groups are shown in Table 1. There were no between-group differences in anthropometric variables at baseline, indicative of the homogeneity of the samples. Post intervention of CrossFit, the IG showed significant before and after on all measured variables (Table 3). The Cohen analysis showed a large effect size in Squat test (*p* < 0.0001; *d* = 2.52), Lunge test (*p* < 0.0001; *d* = 1.61), 20 m shuttle run test (*p* < 0.0001; *d* = 0.86), RESE negative (*p* < 0.0001; *d* = 1.88) and RESE positive (*p* < 0.0001; *d* = 1.9); medium effect size in Push-Up test (*p* < 0.0001; *d* = 0.74). The CG showed no significant differences except for Push-Up test but according to Cohen analysis, the effect size was not relevant (*p* < 0.05; *d* = 0.12). Table 4 shows all the analysis of CG. The greater benefits of a CrossFit program compared to a regular physical exercise program are evident from the comparison of the data reported in Table 3 and Table 4. In particular, the physical tests show greater benefits in CrossFit (IG) practitioners compared to control practicing regular exercise (CG). Further advantages result in the psychological variables from the comparison of the t^0^ < t^1^ scores detected with the RESE Negative and Positive (Table 3 and Table 4).

## 4. Discussion 

Insufficient physical activity can lead to a host of health deficit and maladaptive outcomes [39,40]. The literature suggests that 75% of adolescents and children lack recommended levels of motor activity [41,42]. Schools provide a logical venue to meet the recommended level of physical activity. However, social distancing in the school environment to limit COVID 19 infection has limited this social function. The interruption of training sessions at fitness centers due to the COVID-19 pandemic and the consequent state of "sedentary risk" has been reported in the literature by numerous authors [42,43]. In these studies, statistical evidence shows the increase in damage caused by the lack of physical exercise. They are borne by various organs and systems, cardiovascular, skeletal muscle, respiratory, etc., and also on the psychological conditions (depression and anxiety). The inherent characteristics of CrossFit would make it a viable alternative. An eight-week CrossFit intervention program induced effects on psycho-physical well-being in a group of young adolescents. CrossFit training can be useful as an alternative to classic group sports practiced in a school setting. Interestingly, the RESE results showed improvements on self-efficacy. In 2020, Alemany-Arrebola et al. demonstrated that the worldwide pandemic situation has caused an increase in stress and also showed that a stressful situation (pandemic and confinement) together with a critical event (COVID-19), increases anxiety levels and influences the perception of self-efficacy [44]. Although the connection between self-esteem and regular physical activity is well documented, Dominski et al. (2020) showed a connection between CrossFit training and higher levels of psychological functioning (well-being, affect, body awareness, and self-esteem) [45,46]. Adolescents showed high levels of enjoyment and learning perception after CrossFit practice. Our results are in line with findings by Dominski et al. [46,47]. The effects of the CrossFit protocol on fitness level were significant. The intervention group showed improvements both in strength level (Squat test and Lunge test) and in aerobic level (shuttle run test). On the other side, physical fitness adaptations were already investigated, especially regarding aerobic fitness and body composition [25,48]. CrossFit, just as any other high-intensity training, increases VO_2max_, strength, musculature, and endurance, and decreases lean body mass [19,49]. Physical activity plays an important role for students; recent research showed how physical exercise may enhance academic performance. Rasberry et al. (2011) found positive associations between academic performance and physical activity [50]. Our study proposes CrossFit as a good school exercise activity. As highlighted by this study, in line with the literature, in the Post-CrossFit intervention, the IG showed significant improvements in the values of all the variables considered. The physical variables showed a good increase in the post CrossFit program in comparison with the regular method program despite the adapted training conditions (individualization and distancing). The psychological variables considered, measured with the RESE scale, despite the period of isolation and distancing, have clearly improved as happens in the normal conditions of pre-pandemic training sessions. However, we cannot evaluate if the findings observed would be longer lasting, which is a limitation of the research. Further studies with a larger and more representative sample are needed. Replication of this study, in a public-school setting with more subjects, is therefore recommended. 

## 5. Conclusions

The present work suggests that an 8-week CrossFit intervention program could positively affect the general physical well-being and mental attitude, while also improving the emotional perceived self-efficacy in managing negative affects and in expressing positive emotions in healthy adolescents. Studies have examined the effects of isolation on lifestyles, motor and nutritional habits in the pandemic period. A strong reduction in dynamic behaviors and active lifestyles was highlighted [51,52]. For this reason, any proposal for motor and motivational programs should be tested and applied as an improvement of these psychophysical conditions as we have tried to do with this study.

## Figures and Tables

**Table 1:** Participants’ characteristics.

	IG Intervention Group	CG Control Group	*p* Values
Subjects (n)	15	15	
Age (y)	18.2 ± 0.41	18.33 ± 0.61	ns
Height (cm)	169.66 ± 6.95	172.6 ± 7.16	ns
Weight (Kg)	65.46 ± 6.95	67.66 ± 10.58	ns

**Table 2:** Exercise CrossFit program [37].

Week	Session Parts	Exercises Detail	N° SET/Time
1.1	Warm up	10 russian swings	3 sets
		10 step ups	
		10 lunges	
	Skills/technic	Squats	20 min
		deadlifts	
		burpees	
	WOD AMRAP	20 squats	12 min
		20 deadlifts	
		20 burpees	
	Cool down	Third world sit	5 min
		Lumbar lenghtening	
1.2	Warm up	3 inchworms	3 sets
		10 bear walks	
		6 squats	
	Skills/technic	Back squat	10 min
		Push up	
		box jump	
	WOD AMRAP	10 box jumps	10 min
		10 squats	
		5 push ups	
	Cool down	Hip and gluteal stretches (static)	5 min
2.1	Warm up	3 squats	3 sets
		3 burpees	
		1 shuttle run (20 m)	
	Skills/technic	Goblet squat	
		skipping	
		deadlift	
	WOD AMRAP	1 min goblet squat (wall ball)	3 rounds
		1 min deadlift	
		15 push ups	
		1 shuttle run (20 m)	
	Cool down	Hip and gluteal stretches (static)	5 min
2.2	Warm up	15 burpees	3 sets
		15 box jumps	
		15 squats	
	Skills/technic	Wall ball	10 min
		box jump	
		overhead squat	
	WOD AMRAP	10 wall ball	8 min
		10 box jumps	
		15 push ups	
		run 50 m	
	Cool down	Hip and gluteal stretches (static)	5 min
3.1	Warm up	Hip stretch × 2 (3 min each)	3 sets
		walking lunges (30 m)	
		run (30 m)	
	Skills/technic	Deadlift (barbell)	
		clean (pvc pipe)	
	WOD AMRAP	10 deadlifts	12 min
		5 burpees	
		10 push ups	
	Cool down	Hip and gluteal stretches (static)	5 min
3.2	Warm up	30 step ups	3 sets
		20 kippe	
		10 push ups	
	Skills/technic	clean and jerk (pvc pipe)	10 min
		push press	
	WOD (“Eva”)	800 m run	3 rounds
		double unders	
		30 pull ups	
	Cool down	Hip and gluteal stretches (static)	5 min
4.1	Warm up	30 air squats	3 sets
		20 burpees	
		10 single unders	
	Skills/technic	Thrusters	5 min
	WOD AMRAP	10 thrusters	20 min
		run 50 m	
		10 box jumps	
		run 100 m	
	Cool down	Hip and gluteal stretches (static)	5 min
4.2	Warm up	30 squats	3 sets
		30 squat jumps	
		30 lunges	
	Skills/technic	Push ups	15 min
		double unders	
		handstands	
	WOD (“Annie”)	50-40-30-20-10	FOR TIME
		Single unders	
		sit ups	
	Cool down	Hip and gluteal stretches (static)	5 min
5.1	Warm up	10 kippe	3 sets
		10 jumping jacks	
		10 sit ups	
	Skills/technic	Thrusters	15 min
		Front squat	
		Push press	
	WOD AMRAP	10 burpees	20 min
		10 push press (dumbells)	
		10 thrusters (dumbells)	
		20 sit ups	
	Cool down	Hip and gluteal stretches (static)	5 min
5.2	Warm up	20 m shuttle run	5 sets
	Skills/technic	wall ball cleans	
	WOD AMRAP	10 box jumps	8 min
		8 push ups	
		6 wallball cleans	
		10 GTOH (dumbell)	
		5 sit ups	
	Cool down	Hip and gluteal stretches (static)	5 min
6.1	Warm up	10 squats	3 sets
		10 burpees	
	Skills/technic	Squats and crunch	5 min
	WOD AMRAP	10 wall balls	15 min
		20 m overhead plate	
		20 lunge walks	
	Cool down	Hip and gluteal stretches (static)	5 min
6.2	Warm up	10 leg swings	10 min
		10 burpees	
		10 sit ups	
	Skills/technic	OH squats	5 min
	WOD	10 American Swing	3 rounds
		20 Air squats	
		sprint 50 m	
	Cool down	Hip and gluteal stretches (static)	5 min
7.1	Warm up	30 wall climber	3 sets
		30 swings	
		30 sit ups	
	Skills/technic	Snatch	10 min
	WOD	21 – 15 – 9	FOR TIME
		Snatch	
		Burpees	
	Cool down	Hip and gluteal stretches (static)	10 min
7.2	Warm up	Air bike	10’ + 10’
		Row	
	Skills/technic	Military & Push Press	5 min
	WOD AMRAP (“Danny”)	30 box jumps	20 min
		20 push press	
		30 pull ups	
	Cool down	Pectoral & Dorsal stretching	5 min
8.1	Warm up	30 squats	3 sets
		30 jumping jacks	
		30 burpees	
	Skills/technic	Deadlifts	5 min
	WOD	500 m row	5 rounds
		30 deadlift	
	Cool down	Hip and gluteal stretches (static)	5 min
8.2	Warm up	10 burpees	3 sets
		20 air squats	
		30 jumping jacks	
	Skills/technic	Pull ups	
		TTB or Knees to chest	
	WOD	800 m run	FOR TIME
		400 m row	
		200 m run	
	Cool down	Hip and gluteal stretches (static)	5 min

**Table 3:** Analysis of intervention group.

IG (n15)					
Measure	t^0^	t^1^	Mean of Difference	*p*<	Effect Size *(d)*
BMI	22.64 ± 2.65	22.13 ± 2.58	0.5093	0.0001	0.19
Waist circumference, cm	75.97 ± 8.80	74.27 ± 8.46	1.7	0.0001	0.19
Squat test, rep	28.47 ± 2.36	35.13 ± 2.88	−6.667	0.0001	2.52
Push-up test, rep	8.93 ± 4.43	12.80 ± 5.89	−3.867	0.0001	0.74
Lunge test, rep	31.13 ± 4.61	38.20 ± 4.11	−7.067	0.0001	1.61
20-m shuttle run test, rep	8.40 ± 2.47	10.60 ± 2.59	−2.2	0.0001	0.86
RESE Negative	24.13 ± 4.7	32.33 ± 6.12	−8.2	0.0001	1.88
RESE Positive	25.47 ± 4.91	30.60 ± 3.77	−5.133	0.0001	1.9

**Table 4:** Analysis of control group.

CG (15)					
Measure	t^0^	t^1^	Mean of Difference	*p*<	Effect Size *(d)*
BMI	22.48 ± 2.20	22.45 ± 2.12	0.02667	ns	0.001
Waist circumference, cm	74.87 ± 7.59	74.90 ± 7.53	−0.03333	ns	0.003
Squat test, rep	28.80 ± 2.40	29.40 ± 2.56	−0.6	ns	0.2
Push-up test, rep	9.13 ± 4.2	9.53 ± 4.34	−0.4	0.05	0.12
Lunge test, rep	31.13 ± 5.40	31.40 ± 6.07	−0.2667	ns	0.04
20-m shuttle run test, rep	7.87 ± 2.39	8.07 ± 2.59	−0.2	ns	0.08
RESE Negative	24.27 ± 4.86	24.47 ± 4.77	−0.2	ns	0.04
RESE Positive	23.40 ± 4.40	23.93 ± 3.93	−0.5333	ns	0.12

## Data Availability

Department of Basic Medical Sciences, Neuroscience and Sense Organs, University of Bari “Aldo Moro”, Sport Sciences Section.

## Sample Availability

Samples of the compounds are available from the authors.
